# A micro-elimination approach to addressing hepatitis C in Turkey

**DOI:** 10.1186/s12913-020-5019-8

**Published:** 2020-03-24

**Authors:** Ramazan Idilman, Homie Razavi, Sarah Robbins-Scott, Ulus Salih Akarca, Necati Örmeci, Sabahattin Kaymakoglu, Bilgehan Aygen, Nurdan Tozun, Rahmet Güner, Hurrem Bodur, Jeffrey V. Lazarus

**Affiliations:** 1 Department of Gastroenterology, Ankara University Medical Faculty, Ankara, Turkey; 2 Center for Disease Analysis, Lafayette, CO 80026 USA; 3 Department of Gastroenterology, Ege University Medical Faculty, Izmir, Turkey; 4 Department of Gastroenterology, Istanbul Medical Faculty, Istanbul University, Istanbul, Turkey; 5 Department of Infectious Diseases and Clinical Microbiology, Erciyes University Medical Faculty, Kayseri, Turkey; 6 Department of Internal Medicine and Gastroenterology, Acibadem Mehmet Ali Aydinlar University School of Medicine, Istanbul, Turkey; 7 Department of Infectious Diseases and Clinical Microbiology, Ankara Yildirim Beyazit University, Ataturk Training and Research Hospital, Ankara, Turkey; 8 Department of Infectious Diseases, Ankara Numune Training and Research Hospital, University of Healthcare Sciences, Ankara, Turkey; 9 Barcelona Institute for Global Health (ISGlobal), Hospital Clínic, University of Barcelona, Calle del Rossellón 132, 4th Floor, ES-08036 Barcelona, Spain; 10 Centro de Investigación Biomédica en Red en Epidemiología y Salud Pública (CIBERESP), Instituto de Salud Carlos III, Madrid, Spain

**Keywords:** Hepatitis C, Elimination, Micro-elimination, Modelling, Turkey

## Abstract

**Background:**

In 2016, WHO passed the Global Health Sector Strategy on Viral Hepatitis (GHSS), calling for its elimination by 2030. Two years later, Turkey approved a strategy to reach the WHO targets. This study reports new national prevalence data, breaks it down by subpopulation, and models scenarios to reach HCV elimination.

**Methods:**

Literature was reviewed for estimates of HCV disease burden in Turkey. They were discussed with stakeholders and used as inputs to develop a disease burden model. The infected population was estimated by sequelae for the years 2015–2030. Three scenarios were developed to evaluate the disease burden in Turkey: a Base 2017 scenario, representing the current standard of care in Turkey; an increased treatment scenario, representing the impact of improved access to DAAs; and a WHO targets scenario, which meet the WHO GHSS viral hepatitis targets of a 65% reduction in mortality and 90% diagnosis rate of the infected population by 2030.

**Results:**

At the beginning of 2017, 271,000 viremic infections were estimated. Of these, 58,400 were diagnosed and 10,200 treated. Modelling results showed that, with the current treatment paradigm in Turkey, by 2030 the total number of viremic HCV infections would decline by 35%, while liver-related deaths, hepatocellular carcinoma (HCC), and decompensated cirrhosis would decrease by 10–25%. In the *increased treatment scenario*, by 2030 viremic HCV infections would decrease by 50%; liver-related deaths, HCC and decompensated cirrhosis would decrease by 45–70%. In the *WHO targets scenario*, HCV infections would decrease by 80%; sequelae would decrease by 80–85%. Data on disease burden in micro-elimination target subpopulations are largely unavailable.

**Conclusions:**

To meet the WHO Global Health Sector Strategy targets for the elimination of HCV, Turkey needs to increase treatment. Better data are needed as well as countrywide access to DAAs.

## Background

The introduction of all-oral second-generation direct-acting antiviral (DAA) drugs in 2013 dramatically changed treatment paradigms and outcomes of hepatitis C virus (HCV) infection worldwide. Very high sustained virologic response (SVR) rates (≥95%) can be achieved with 8–12 weeks of DAA treatment [1]. These high treatment success rates led the World Health Organization (WHO) to call for the elimination of viral hepatitis in their 2016 *Global health sector strategy on viral hepatitis* (GHSS) and set targets for the elimination of HCV infection by 2030 – a 90% reduction in new HCV infections and a 65% reduction in mortality due to HCV [2].

Eliminating HCV countrywide requires the inclusion of diverse stakeholders – governmental authorities at the national, subnational, and local levels, associations of health-care providers, patients, and representatives of at-risk populations. It calls for complex planning in terms of public affairs, and the need for human and financial resources that may not be readily available [3]. Before attempting nationwide elimination, breaking down national elimination goals into smaller, achievable goals for individual population segments may be more realistic [3]. Such micro-elimination strategies for selected populations are easier to develop and implement; targets can be achieved in a shorter period of time; and fewer financial resources are required at the outset. Further, they encourage health-care providers and other stakeholders to develop a broader programme to achieve the WHO elimination targets following the success of micro-elimination plans.

The prevalence of HCV infection in Turkey ranged from 275,000 (0.5%) to 494,000 (0.96%) in nationwide epidemiologic studies in 2009–10 [4, 5]. Only 15,000–20,000 patients have been treated with DAAs from mid-2016 to mid-2018.

Two second-generation DAA regimens were approved by the Turkish Ministry of Health in 2015 and treatment with these has been reimbursed since 2016. An HCV-infected patient who has been previously treated with other antiviral treatment is also eligible for reimbursement of DAA treatment without any limitation. However, to start DAA treatment, a treatment-naive HCV-infected patient is required to be at least in fibrosis stage F1 (Ishak scoring system) documented by a liver biopsy, a procedure not favored by most patients. In addition, the national reimbursement authority reimburses DAAs prescribed only by infectious disease or gastroenterology specialists from tertiary referral hospitals, which are only in 41 cities of Turkey, greatly limiting access. Treatment is delivered to patients only via pharmacies in these hospitals.

The Turkish Ministry of Health published a highly comprehensive national HCV elimination plan in October 2018, but how it will be implemented is unknown [6]. To examine the feasibility of achieving the WHO targets, including through a national micro-elimination programme, reliable disease burden estimates are needed [7] in the general population and in target subpopulations. This study reports new national prevalence data and breaks it down by subpopulation, employing a micro-elimination approach, and models the feasibility of eliminating viremic HCV by 2030.

## Methods

In December 2017–January 2018, a literature search was conducted to identify estimates of hepatitis C prevalence, viremia, genotype distribution, leave in diagnosis, and treatment rates in Turkey. A Delphi process was then initiated in January 2018 with a team of country experts to assess the body of evidence and reach consensus on all inputs of the model and sub-population level estimates. The group consisted of 44 physicians representing infectious disease and gastroenterological professional associations (*see* the Acknowledgements section), and four meetings were held in Ankara, Istanbul and Izmir during the study process.

### Disease burden model

An Excel-based Markov HCV disease burden model was developed [8]. Population, mortality, and historical data specific to Turkey were used to calibrate the model, and the infected population was estimated by HCV sequelae for the years 2015–2030 (Table 1). The model reflects the natural history of HCV infection, in that it begins with the annual number of acute infections that progress to chronic HCV, after considering spontaneous clearance. The progression of these new cases is followed along with chronic infections from the previous year (all by age and sex), accounting for treatment, cure, and background mortality. When available, reported or calculated annual estimates of new infections are used. However, these data were not available in Turkey and, thus, new cases by age and sex were back-calculated using known prevalence in a given year. Considering that the model calculated all-cause mortality, liver-related mortality, and cured cases, Solver, an optimization add-in by Excel, was used to determine the average number of new infections per year dating back to 1950. Then, an annual relative incidence value was used to describe the change in the number of new infections from 1950 until the year of known HCV prevalence.

**Table 1:** **Estimations used to model the burden of HCV in Turkey**

**a. Base 2017 scenario**						
	**2015**	**2016**	**2017 & 2018**	**2018**	**2019**	**≥2020**
**Treated**	4200	5600	10,200	9500	8800	5600
**Newly diagnosed**	5500	5500	5500	5500	5500	5500
**Fibrosis stage**	≥F0	≥F3	≥F1	≥F1	≥F1	≥F1
**Treated age**	15–79	15–79	15–79	15–79	15–79	15–79
**SVR**	49%	97%	99%	99%	99%	99%
**b. Increased treatment scenario**						
	**2015**	**2016**	**2017 & 2018**	**2019**	**2020–2024**	**≥2025**
**Treated**	4200	5600	10,200	11,000	11,000	11,000
**Newly diagnosed**	5500	5500	5500	5500	5500	5500
**Fibrosis stage**	≥F0	≥F3	≥F1	≥F0	≥F0	≥F0
**Treated age**	15–79	15–79	15–79	15–79	15–79	15–79
**SVR**	49%	97%	99%	99%	99%	99%
**c. WHO targets scenario**						
	**2015**	**2016**	**2017 & 2018**	**2019**	**2021–2024**	**≥2025**
**Treated**	4200	5600	10,200	15,000	16,000	16,000
**Newly diagnosed**	5500	5500	5500	6000	18,000	18,000
**Fibrosis stage**	≥F0	≥F3	≥F1	≥F1	≥F0	≥F0
**Treated age**	15–79	15–79	15–79	15–79	15–79	15–79
**SVR**	49%	97%	99%	99%	99%	99%

Discussions with experts were used to inform the trend in new infections, considering risk factors such as blood transfusion and injection drug use. After these calculations were complete, the age and sex distribution of acute infections were calculated to match the age and sex distribution of the known prevalent population and linearly trended in five-year increments. The total number of cases by HCV sequelae was calculated by multiplying the total number of cases at a particular stage by a progression rate, adjusting for ageing, all-cause mortality, and cured in a given year. All cause mortality rates by age and sex were gathered from the United Nations mortality database and adjusted for incremental increase in mortality due to injection drug use and transfusion. The number of active people who inject drugs (PWID) and HCV prevalence among PWID was gathered through published studies and divided by the total HCV infected population in order to estimate the proportion of all viremic HCV infections among active PWID.

### Inputs for estimating the disease burden

There were an estimated 396,400 cases with anti-HCV positivity (0.52% prevalence) in 2013 [9]. Applying a viremic prevalence rate of 71.7% [9], 284,200 individuals were estimated to be chronically infected with HCV in 2013 in the general Turkish population, resulting in a viremic prevalence of 0.37%. The model assumes that 86.7% of infections are genotype 1 (8.8% G1a, 70.2% G1b, and 7.7% G1 other) and 6.4% are genotype 3, based on a study of approximately 1,000 patients [10]. The age and sex distribution of the infected population was taken from a study conducted in 2009–2010 in 23 urban and rural areas of Turkey [4]. About 59,700 viremic infections were diagnosed as of 2017, and of those, 5,500 viremic patients are newly diagnosed each year (expert input). Industry data report that 5,600 patients were treated in 2016; all had ≥F3 fibrosis. In 2017, treatment eligibility was expanded to patients ≥F1, allowing for 10,200 patients to be treated in that year. Literature sources informed rates of infection associated with injecting drug use and blood transfusion [11], two HCV risk factors associated with a higher mortality. These values were agreed upon as the best acceptable figures.

Once the current HCV disease burden was estimated, strategies to minimize the future burden were analyzed. Two intervention scenarios that expand access and increase rates of care above current levels were compared to a historical base case through the year 2030.

#### Scenarios

The “Base 2017” scenario represents the current treatment paradigm in Turkey (Table 1a). Without additional efforts to identify new cases and expand treatment eligibility, it was assumed that a diminishing pool of treatment-eligible patients would result in a 50% decline in the annual number of patients treated by 2020. After 2020, the annual number of treated patients is assumed to remain constant. Patients aged 17–91 years were eligible for treatment, and the SVR rate was estimated to be 99% in 2018 [12].

An “Increased Treatment” scenario was created to assess the impact of improved access to DAAs in the coming years (Table 1b). Treatment was expanded to 11,000 patients annually beginning in 2019, regardless of fibrosis stage. However, as no national screening strategies have been developed for Turkey, the annual number of newly diagnosed patients was assumed to remain constant. Because of this, the model would run out of eligible patients by 2027. Eligible treatment age and SVR rates remained the same as the Base 2017 scenario.

A third scenario was developed in order to meet the WHO GHSS viral hepatitis targets of a 65% reduction in mortality and 90% diagnosis rate of the infected population by 2030 [2]. To do this, the number of treated patients was increased to 16,000 annually starting in 2021 (Table 1c). The number of diagnosed patients was also increased to 18,000 annually by the same year. Incidence was reduced starting in 2021 to achieve an overall 90% reduction in annual new infections by 2030.

### Estimating the burden of prevalent HCV infection in priority subpopulations targeted for micro-elimination

Ways to incorporate micro-elimination targets within this wider framework of national elimination strategies were proposed through the series of expert meetings. In Turkey, the potential priority at-risk populations that can be targeted for HCV micro-elimination could be categorized into three broad groups: (i) patients at high risk who are already in the health-care system; (ii) people at high risk due to demographic characteristics; and (iii) people with high-risk behaviours (Table 2).

**Table 2:** Potential prioritized at-risk populations that can be selected for micro-elimination in Turkey

1. Patients with high risk and already in the health-care system	
a. Patients with advanced liver disease	
b. Patients with advanced chronic kidney disease	
c. Patients with certain hematologic diseases (i.e. haemophilia, thalassemia)	
d. Transplant recipients	
e. Persons diagnosed with HCV but not treated yet	
2. People at high risk due to demographic characteristics	
a. People who were born before 1960	
b. People from certain geographic areas	
3. People with high-risk behaviours	
a. People who inject drugs	
b. Prisoners	

## Results

### HCV disease burden in special populations

Through discussions with over 40 experts at four meetings across the country, coupled with a literature review, the following sub-population level estimates were projected.

#### Patients at high risk who are already in the health-care system

##### Patients with advanced liver disease

Advanced liver disease is defined by significant fibrosis (>F3 assessed by either APRI score > 1.5, FIB-4 > 3.2, transient elastography > 9.5 kPa or biopsy > METAVIR stage F3). Estimates of the disease burden showed a compensated cirrhosis prevalence of 13%, and a decompensated cirrhosis prevalence of 1.6% among viremic patients [13].

##### Patients with advanced chronic kidney disease

No published data are available on the number of patients with HCV who undergo dialysis [14–17]. However, the Turkish Society of Nephrology reported that almost 60,000 patients undergo some kind of dialysis routinely in the 860 dialysis centres in Turkey and, based on the data from 72 centres, 5.5% of those undergoing hemodialysis are positive for anti-HCV [18].

##### Patients with certain inherited blood disorders (i.e. hemophilia, thalassemia)

In 2014, there were 6,150 patients with hemophilia, Von Willebrand disease or other bleeding disorders, and at least 4,500 patients with beta-thalassemia in 2007 [19, 20]. How many of these are HCV-infected is not known. In 2005, 14 of 70 patients (20%) with thalassemia major were anti-HCV positive, five (7.1%) were HCV RNA positive [21]. A publication in 2018 stated that among 53 patients with thalassemia major, two were anti-HCV positive – Syrians, who had immigrated from Syria [22]. A study in 2017 among 1,270 patients in 33 centres found that 21.7% had a history of blood transfusion [23].

##### Transplant recipients and patients on immune-suppressive treatment

No data are available on the number infected with HCV among this population. Ministry of Health data show that HCV prevalence was 9–11% among transplant recipients between 2011 and 2015.

##### Persons who have already been diagnosed with HCV but not treated yet

There are no published data on the number of patients who have been diagnosed with HCV but not treated yet.

#### People at high risk due to demographic characteristics

##### People who were born before 1960 [24]

There are about 19.5 million people in this group, but it is not known how many are currently infected with HCV. People who received blood or blood products or underwent an organ transplantation before 1996 may also be included as a risk group.

##### People from certain geographic areas

Living in specific regions of south-eastern Turkey is associated with a 2.1-fold increase in the chances of being anti-HCV positive compared to living in other areas of Turkey [4]. Another study showed 11% anti-HCV positivity in Nevsehir in the region of Cappadocia [25].

#### People with high-risk behaviors

##### People who inject drugs

The prevalence of anti-HCV positivity was estimated to be around 40% in 2015 [26].

##### Prisoners

Anti-HCV positivity was 17.7% among prisoners [27].

#### Estimates of disease burden

Additionally, using the aforementioned disease burden model, the following general population level estimates were projected and the impact of three different management scenarios assessed.

**Fig. 1:**
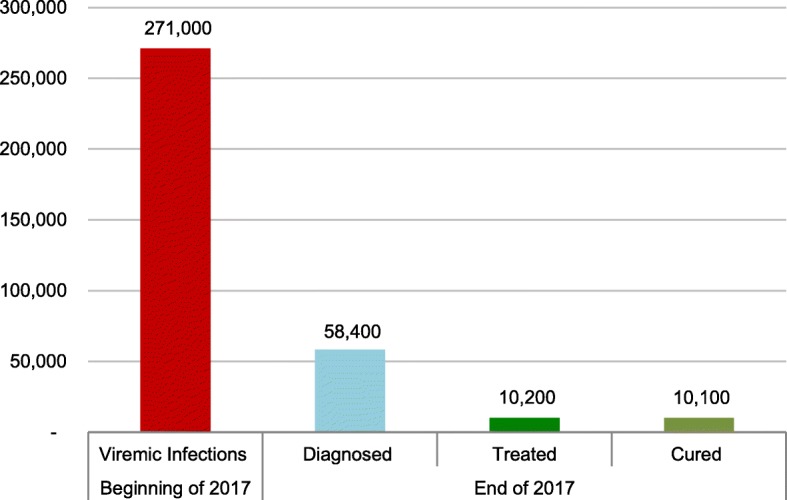
The hepatitis C cascade of care, including the total number of viremic infections, the number of diagnosed patients, and the number of patients treated and cured, in Turkey in 2017

The number of viremic infections estimated at the beginning of 2017 was 271,000. Of these, 58,400 were diagnosed and 10,200 treated. At a 99% SVR rate, 10,100 of the 10,200 treated were estimated to be cured (Fig. 1).

Regarding HCV prevalence by age cohort, the number of HCV infections started rising from the age of 15 years, peaked at 35–44 years and gradually declined thereafter.

#### Disease burden scenarios

##### Base scenario

**Fig. 2:**
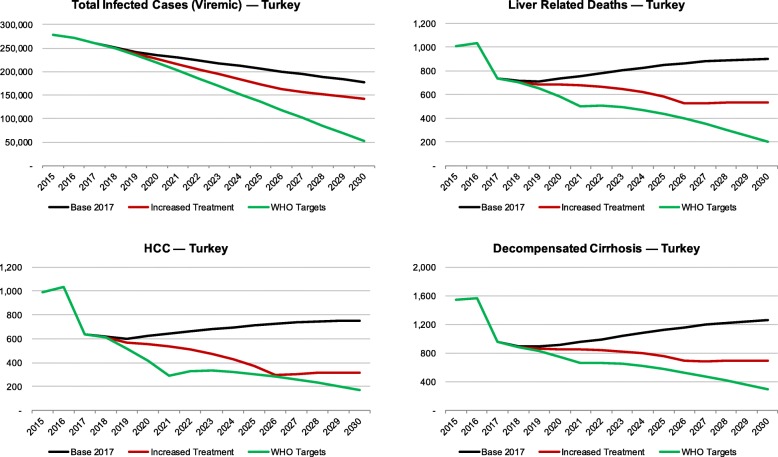
Total infected cases, liver-related deaths, prevalent HCC and prevalent decompensated cirrhosis in Turkey, 2015–2030

By 2030, the total number of viremic HCV infections would decline by 35%. The number of HCV infected people with hepatocellular carcinoma (HCC) and decompensated cirrhosis cases would decrease by 10–25% by the same year. (Fig. 2 and Table 3).

**Table 3:** Total infected cases, decompensated cirrhosis, HCC and liver-related deaths in Turkey, by scenario, 2015 and 2030

	2015	2030
Total viremic infections		
Base	278,000	177,000
Increased treatment	278,000	142,000
WHO targets	278,000	52,000
Prevalent Decompensated cirrhosis		
Base	1500	1300
Increased treatment	1500	700
WHO targets	1500	290
Prevalent Hepatocellular carcinoma		
Base	990	750
Increased treatment	990	320
WHO targets	990	170
Liver-related deaths		
Base	1000	900
Increased treatment	1000	530
WHO targets	1000	200

##### Increased treatment scenario

By 2030, the total number of viremic HCV cases would decrease by 50%; and liver-related deaths, prevalent HCC and prevalent decompensated cirrhosis would decrease by 45–70% (Fig. 2 and Table 3).

##### Scenario to meet the WHO GHSS targets

The total number of viremic HCV infections would decrease by 80%; liver-related deaths, prevalent HCC and prevalent decompensated cirrhosis would decrease by 80–85% (Fig. 2 and Table 3).

## Discussion

The availability of DAA therapy with its high SVR rates has allowed Turkey to embark on reaching the WHO targets for the elimination of viral hepatitis as a public health threat by 2030. The cost of treatment in Turkey is a major bottleneck to the large scale roll-out in the country, despite DAA therapy being shown to be cost-effective in other countries, when delivered at scale and in combination with prevention and screening strategies [28–30]. In Turkey, the average annual cost (excluding hepatitis C drug costs) of a patient with chronic hepatitis C, compensated cirrhosis, decompensated cirrhosis, HCC and liver transplant is US$ 446.83, US$ 577.56, US$ 1984.39, US$ 2474.15, US$ 42,469.27, respectively [31]. To reach the WHO targets, early diagnosis and treatment are crucial and cost-saving, as inaction or severe disease costs significantly more. This study found that in all three of the modelled treatment paradigms, by 2030, the total number of viremic infections, liver-related deaths, HCC and decompensated cirrhosis would decrease, which would be highest if the WHO targets are achieved.

If a micro-elimination approach focuses on patients already in the health system, screening, treatment and prevention interventions can often be provided more easily, without additional burden on the health system. Furthermore, as in other parts of the world, models of care that tailor to the needs of sub-populations should be considered whenever possible [32], if elimination is to be achieved.

### Addressing micro-elimination in subpopulations

#### Patients at high risk who are already in the health-care system

##### Patients with advanced liver disease

Such patients have probably already been diagnosed and are being followed up regularly [5]. As these patients are evaluated at least once a year by a gastroenterologist, they could easily be also evaluated for HCV.

##### Patients with advanced chronic kidney disease

Turkey has a well-organized and fully reimbursed hemodialysis/peritoneal dialysis system for patients with chronic kidney disease and almost all patients are followed up by a dedicated dialysis centre. These centres can therefore be included in a comprehensive evaluation and yearly HCV screening programme of both current and prospective patients.

##### Patients with certain inherited blood disorders (i.e. hemophilia, thalassemia)

Due to their requirement for blood or blood component transfusion, these patients are vulnerable to acquiring HCV. As they are closely followed up by certain hospitals and/or special treatment centres in Turkey, their HCV status can be evaluated by including these institutions in plans for micro-elimination.

##### Transplant recipients and patients on immune-suppressive treatment

During 2016, of 4,906 transplants nationally, approximately 1,300–1,400 were liver transplants. The number is increasing each year [33]. Such patients are subject to severe complications of infection. The medical condition of these patients and current reimbursement legislation require routine follow up by the relevant medical specialty departments and their records are kept properly. A plan that focuses on the HCV status of patients in these transplantation centres can be easily implemented.

##### Persons who have already been diagnosed with HCV but not treated yet

Big dentistry clinics and many general health-care settings routinely evaluate patients for certain infections preoperatively, but often do not adequately refer them. Surgeons, dentists and anaesthesiologists can be trained to detect such patients, and the hospital information technology system improved to institute a warning for patients who test positive or provide an automatic computer-based referral to the relevant clinics.

Since the mid-1990s, all blood banks in Turkey routinely evaluate all blood donations for certain infections. Anti-HCV-positive donors are informed about their situation, but there is no follow-up system. Hence, most of them are not admitted to the relevant specialty clinics. Blood bank personnel can be trained, and a referral and follow-up system developed for such donors. Hospital and blood bank records can also be evaluated retrospectively to identify patients with a positive anti-HCV test result. A dedicated team in each hospital or blood bank can contact these patients and organize further testing and referral procedures.

#### People at high risk due to demographic characteristics

##### People who were born before 1960

The use of non-disposable medical equipment was common in Turkey before 1990 and routine HCV screening for all blood and blood products was not mandatory before the mid-1990s. Therefore, those who underwent any kind of medical procedure, got vaccination, or received any kind of blood or blood product before the mid-1990s may have been exposed to HCV. People born before 1970 were clearly at higher risk for acquiring HCV infection. Local data from Turkey also confirmed this, which revealed that the mean age of patients with positive anti-HCV was 49 years in 2010 [4]. Therefore, a national plan that includes evaluation of the HCV status of people who were born before 1960 and/or have high alanine aminotransferase/aspartate aminotransferase (ALT/AST) levels would identify many patients who suffer from undiagnosed HCV and its complications.

##### People from certain geographic areas

Living in south-eastern Turkey is associated with a 2.1-fold increase in the chances of being anti-HCV positive compared to living in other areas of Turkey [4]. The Cappadocia region also has a higher prevalence of HCV. A national HCV screening strategy that targets HCV-prevalent areas, such as Kirikhan district in Hatay city, can identify those who are currently infected with HCV.

#### People with high-risk behaviours

##### People who inject drugs

In Turkey, specialized addiction centres (AMATEMs) provide inpatient and outpatient health-care services to people who inject drugs. Nationally, around 12,000 people were admitted to these in 2015. The prevalence of anti-HCV positivity among people who inject drugs was estimated to be around 40% in 2015 [26].

The benefits of providing training to health-care professionals at AMATEMs and defining systems for referral and access to treatment for anti-HCV-positive patients at these centres has been described previously [34]. Freely accessible treatment of HCV in people who inject drugs can prevent HCV transmission to uninfected people and reinfection among those treated.

##### Prisoners

Prisoners are a vulnerable population and are among the potential priority at-risk populations for micro-elimination [35]. The continued use of drugs and shared syringes, getting new tattoos, and other incidents that cause contact with blood increase the risk of blood-borne infections among prisoners [27]. A national plan formulated jointly by both the Ministry of Health and Ministry of Justice should include training of health-care providers in prisons, screening of prisoners and defining the methodology to access treatment.

### Barriers to improving access to direct-acting antivirals in Turkey

The current reimbursement conditions for DAAs are flexible; however, treatment can be initiated only by gastroenterologists or infectious disease specialists in authorized tertiary hospitals in 41 cities of Turkey. Based on the outcomes of the meetings, it was recommended that all gastroenterologists and infectious disease specialists, regardless of hospital type and city, be authorized to start DAA treatment by the national reimbursement authority.

The majority of antiviral treatment-naive patients living with HCV are at least at the F1 stage of fibrosis at the time of diagnosis. Therefore, the requirement for classifying antiviral treatment-naive patients by fibrosis type with a documented biopsy has no practical benefit. Moreover, the interventional nature of the biopsy may cause some medical complications and patient discomfort [36]. This requirement for accessing treatment should be removed, as in most countries of the world [37].

In addition to the aforementioned barriers to improving HCV treatment access in Turkey, the lack of epidemiological data available in the country hinders proper reporting and surveillance efforts. One solution would be to simplify the cascade of care by using the consensus definition to standardize HCV reporting and monitor elimination effort progress across the country and in all sub-populations [38].

### Limitations

Our results had several methodological limitations. The model was calibrated with the best available data, supplemented by a Delphi process to gain agreement on all inputs by national experts. The actual number of total infections diagnosed and treated may be different. In addition, the model does not take into account the impact (either on disease burden or economically) of reinfection or comorbidities. More so, though this model does not dynamically estimate new infections or reinfections, the high treatment rate required of the specified elimination scenario, coupled with reduced treatment restrictions and prevention programs aimed at high-risk groups, exceed the treated proportion specified in other dynamic models. Lastly, the increases in treatment and diagnostic levels may not be realistic, given the current healthcare and economic constraints in Turkey. The modelled scenarios use inputs for the number of diagnosed cases necessary to treat the indicated number of patients at a sustained level. Finding undiagnosed cases will become more difficult as the diagnosis rate increases. Finally, a sensitivity analysis was not conducted in this study. The uncertainty in HCV prevalence dominates all previous sensitivity analyses and accounted for the majority of the variance. However, the decisions and recommendations here would still be the same. Thus, for the purposes of supporting decisionmaking by the policy-makers, only the deterministic results were presented.

## Conclusions

Turkey has the potential to achieve the WHO HCV elimination targets if it greatly increases the number of individuals diagnosed and treated. The results of modelling the burden of HCV in Turkey and contextualizing infected subpopulations in the overall infected population showed the value of the micro-elimination approach in reaching the WHO elimination targets. However, better reported data and estimates are needed countrywide. Further, all regions of the country should have access to DAAs, and the recent removal of the requirement for a liver biopsy to initiate treatment should facilitate the process. These investments will pay off by saving lives and averting costly interventions that will occur in the future if the WHO 2030 targets are not met.

## Data Availability

Additional data are available upon request to JVL.

## Abbreviations

DAA: Direct-acting antiviral
HCC: Hepatocellular carcinoma
HCV: Hepatitis C virus
SVR: Sustained virologic response
WHO: World Health Organization
